# Clinico-Histomorphological and Immunohistochemical Profile of Anaplastic Pleomorphic Xanthoastrocytoma: Report of Five Cases and Review of Literature

**Published:** 2018-10-01

**Authors:** Prita Pradhan, Biswajit Dey, Bheemanathi Hanuman Srinivas, Sajini Elizabeth Jacob, Roopesh Kumar Vadivel Rathakrishnan

**Affiliations:** 1 Department of Pathology, Jawaharlal Institute of Postgraduate Medical Education and Research, Puducherry, India; 2 Department of Pathology, Andaman and Nicobar Islands Institute of Medical Sciences, Port Blair, India; 3 Department of Neurosurgery, Jawaharlal Institute of Postgraduate Medical Education and Research, Puducherry, India

**Keywords:** Astrocytoma, Anaplasia, Mitosis, Immunohistochemistry, Ki 67

## Abstract

**Background:** Pleomorphic xanthoastrocytoma is a rare tumour of children and young adults, particularly for those with features of anaplasia.

**Materials and Methods** **:** This retrospective study comprises five cases of anaplastic pleomorphic xanthoastrocytomas diagnosed over a period of 4 years in a tertiary care institute. A detailed clinicopathological and immunohistochemical profile of the tumours were noted from the hospital database.

**Results: **Five cases of anaplastic pleomorphic xanthoastrocytomas were evaluated for their clinicoradiological, histomorphological as well as immunohistochemical findings, which included 3 females and 2 males, with age range of 11-40 years and a mean age at presentation of 22 years. Histologically a solid cystic biphasic tumour with moderate to high cellularity, spindled pleomorphic astrocytes, hyperchromatic nuclei showing moderate to marked atypia, intranuclear inclusions, ≥5 mitoses per 10 high power fields, with evidence of necrosis and atypical mitoses was noted. One of the cases showed transformation into glioblastoma with evidence of spinal metastasis on follow-up. The tumours expressed both glial as well as neuronal markers with expression of CD34 with increased Ki 67 ranging between 5-20%.

**Conclusion: **It was concluded that PXA, a low-grade glioneuronal tumour, can show odd site presentation, marked pleomorphism, increased mitosis, atypical mitoses and increased Ki 67 when associated with features of anaplasia. An appropriate panel of immunohistochemical markers in conjunction with a detailed evaluation of histomorphological features and clinicoradiological information are useful for its diagnosis.

## Introduction

 Pleomorphic xanthoastrocytoma (PXA) is a rare tumour of children and constitutes less than 1% of all astrocytic glial neoplasms^1^^,^^2^. PXA is a World Health Organisation (WHO) grade II tumour with favourable prognosis^3^. It is now known that 15-20% of PXA show anaplastic features with evidence of >5/10 HPF mitotic figures and focal necrosis. These cases are now labelled as anaplastic pleomorphic xanthoastrocytoma (APXA), WHO grade III and are recognised as a distinct entity according to the newer classification for the central nervous system tumours (2016) proposed by the World Health Organisation^3^. Anaplastic PXA (APXA) is either primary i.e. de novo or secondary i.e. anaplastic transformation in recurrent conventional PXA^4^. APXA has a poor prognosis and rarely can undergo transformation to glioblastoma^5^. All the cases of APXA were evaluated for their clinicoradiological, histomorphological and immunohistochemical (IHC) findings. 

## MATERIALS AND METHODS

 This retrospective study included 5 cases of APXAs diagnosed over a period of 4 years (January 2012 to December 2016) in a tertiary care institute. A detailed clinicopathological and IHC profile of the tumours were noted from the hospital database.

All the samples were fixed in 10% neutral buffered formalin and embedded in paraffin. Staining was done with haematoxylin and eosin. The archival hematoxylin and eosin slides were reviewed by the authors. IHC staining for monoclonal antibodies Glial Fibrillary Acid Protein (GFAP), S 100, Neuron Specific Enolase (NSE), CD 34, Synaptophysin and Ki67 were done by Avidin Biotin peroxidase method with pre-treatment by microwave heating. 

All histopathological slides made from the paraffin blocks and submitted to the department of pathology were reviewed in this study. Informed consent was obtained from the patents or their relatives for use of data for publication purpose. As this retrospective study had no interventions other than the standard care, permission from the institutional review board was not obtained.

## Results

 There were 5 cases of APXA diagnosed based on histopathological and immunohistochemical findings. The age of the patients ranged from 11 years to 40 years with a mean age of 22 years. There were 3 females and 2 males with M: F ratio of 3:2. All the 5 patients had varied clinical presentations with duration of symptoms ranging from 1 week to 1 month. The sites and the corresponding clinicoradiological diagnosis are summarised in Table 1.

**Table 1:** Site of lesion with the corresponding clinicoradiologic and histopathologic diagnoses

**Case**	**Site**	**Clinicoradiologic ** **diagnosis**	**Final histopathologic ** **impression**
1	Left occipito parietal region	High grade glioma	Anaplastic PXA
2	Intramedullary (D8-L1) lesion	Astrocytoma/ependym oma	Anaplastic PXA
3	Left temporal region	Low grade glioma	Anaplastic PXA
4	Right frontal area	High grade glioma	Anaplastic PXA
5	Right frontal area	High grade glioma	Anaplastic PXA

## Case presentation

**Case 1**

An 11- year-old male child presented with the complaints of repeated vomiting and headache for a period of one month. On examination, the child was found to have right-sided homonymous hemianopia along with papilledema. Magnetic resonance imaging (MRI) revealed a ring enhancing cystic mass lesion with perilesional edema in the left occipito-parietal region. Craniotomy was done with a pre-operative diagnosis of high-grade glioma. Intra-operatively, a thin but vascular cyst containing yellowish fluid was identified with no clear plane between tumour and normal brain tissue. Tumour was excised. Histomorphological examination and immunohistochemistry findings are summarised in Table 2.

**Table 2:** Histomorphologic and immunohistochemical findings of the original neoplasms

**Histologic features**		**Case 1**	**Case 2**	**Case 3**	**Case 4**	**Case 5**
Cellularity		Moderate	Moderate	Variable	High	High
Predominant cell type		Spindle cells	Spindle cells	Pleomorphic bizarre cells	Pleomorphic bizarre cells	Pleomorphic bizarre cells
Mono-/ Bi-/ Multi- nucleated cells		Present	Present	Present	Present	Present
Pleomorphic hyperchromatic nucleus		Present	Present	Present	Present	Present
Nuclear atypia		Moderate	Mild to moderate	Moderate to marked	Moderate to marked	Moderate to marked
Intranuclear inclusions		Present	Present	Present	Present	Present
Mitotic figures		5/10 HPF	5/10 HPF	6/10 HPF	8/ 10 HPF	8/ 10 HPF
Atypical mitoses		Absent	Absent	Absent	Present	Absent
Necrosis		Absent	Focal	Absent	Absent	Absent
Xanthoma cells		Present	Present	Present	Present	Present
Eosinophilic granular bodies		Present	Present	Present	Present	Present
Microvascular proliferation		Absent	Absent	Absent	Absent	Present
Perivascular lymphocytes		Present	Present	Present	Present	Present
Hemorrhage and hemosiderin laden macrophages		Absent	Absent	Present	Absent	Absent
Meningeal invasion		Present	Present	Present	Present	Present
IHC	GFAP	+	+	+	+	+
	S100	+	+	+	+	+
	NSE	focal+	+	-	Focal+	-
	CD34	focal+	-	-	Focal+	-
	Syn	-	+	-	-	focal+
	Ki67	6%	5%	10-15%	20%	18%

**Case 2**

**Figure 1:**
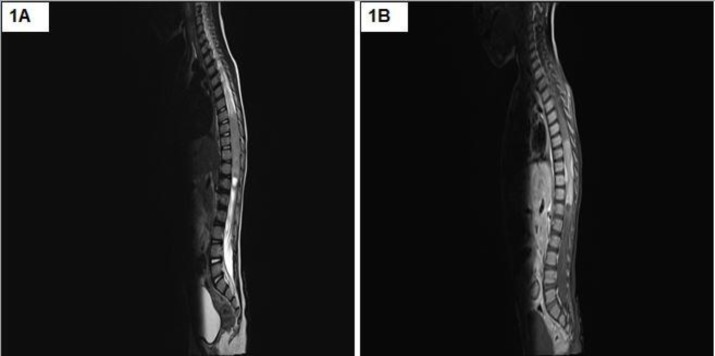
MRI showing an intramedullary mass lesion extending from D8-L1 which was hypointense on T1 (1A) and hyperintense on T2 (1B) with a cyst at lower pole

**Figure 2:**
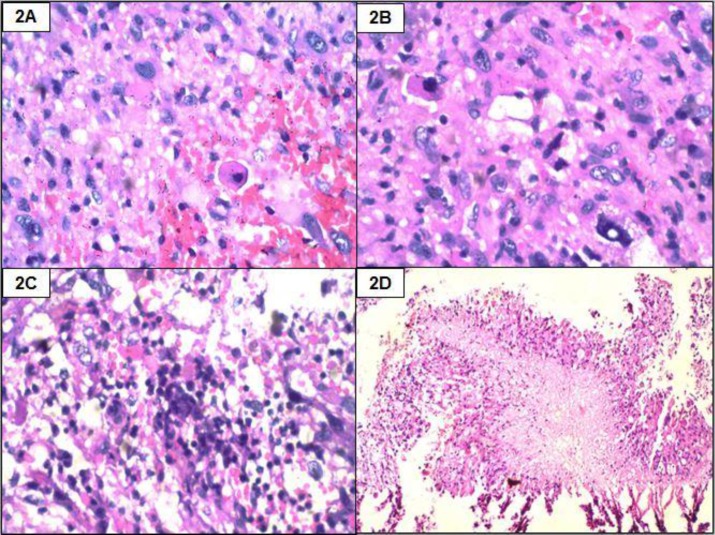
Histopathology showin g moderately cellular tumour (2A, H & E, 400x) with xanthoma cells and eosinophilic granular bodies (2B, H & E, 400x). Binucleated cells (2C, H & E, 100x) and focal necrosis seen. (2D, H & E, 40x).

**Figure 3:**
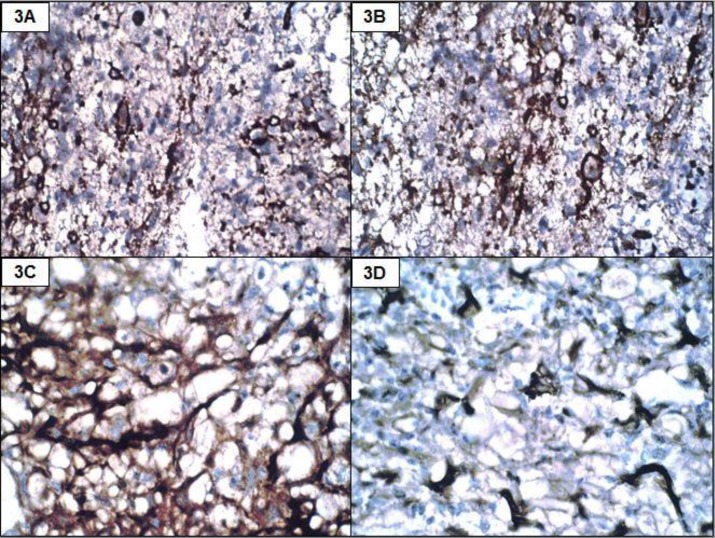
Positivity with GFAP (3A, IHC, 100x), S100 (3B, IHC, 100x), NSE (3C, IHC, 400x) and synaptophysin (3D, IHC, 400x)

**Figure 4:**
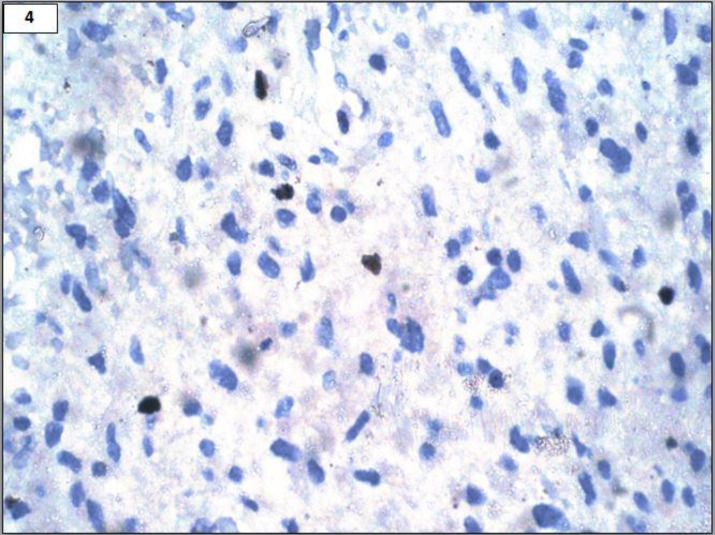
Ki67 labelling 5% (IHC, 400x)

A 22-year-old male presented with acute onset paraplegia with mean duration of one month. MRI revealed an intramedullary contrast enhancement of mass lesion extending from D8-L1. It was hypointense on T1 and hyperintense on T2 with a cyst at lower pole (Figures 1A and 1B). Intra-operatively, a grey, soft and suckable mass was found towards the left. Though no clear tissue plane could be made out, the normal cord tissue appeared thinned out. Histomorphologic examination and immunohistochemistry findings are summarised in Table 2, Figures 2, 3 and 4.

**Case 3**

A 40-year-old female presented with complaints of urinary incontinence and vomiting for one week. On MRI, a well-circumscribed, contrast enhancing, dural-based mass measuring 6x5cm in the left temporal was seen. Craniotomy was done. Intra-operatively the tumour was found to be very vascular. The tumour was completely excised. Histomorphologic examination and immunohistochemistry findings are summarised in Table 2.

**Case 4**

A 19- year-old male presented with headache and two episodes of generalised tonic clonic seizures over one month. On examination the vitals were found to be stable. He was conscious and oriented. There were no focal neurological deficits. MRI highlighted a cystic lesion with enhancing nodular component measuring 5X5 cm within the right frontal area near the motor cortex. Craniotomy and decompression of lesion were performed. Histomorphologic examination and immunohistochemistry findings are summarised in Table 2.

**Case 5**

**Figure 5:**
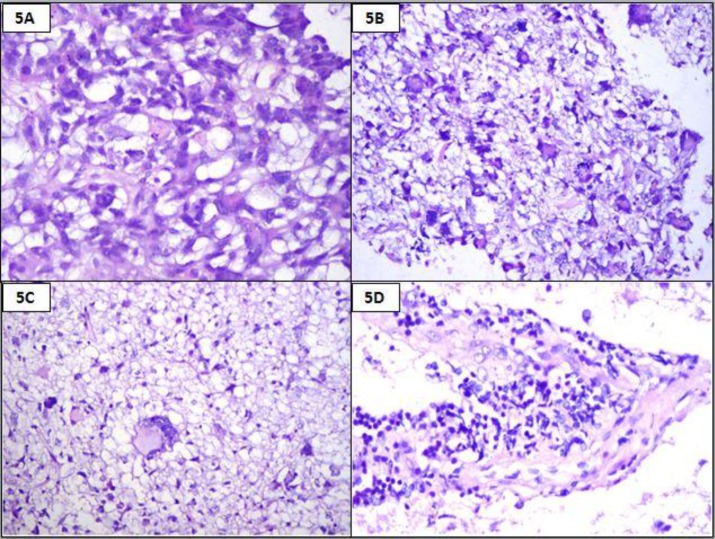
Histopathology showing cellular tumour with xanthoma cells (5A, H & E, 400x) and multinucleated cells (5B and 5C, H & E, 100x). Perivascular lymphocytes seen (5D, H & E, 100x)

**Figure 6:**
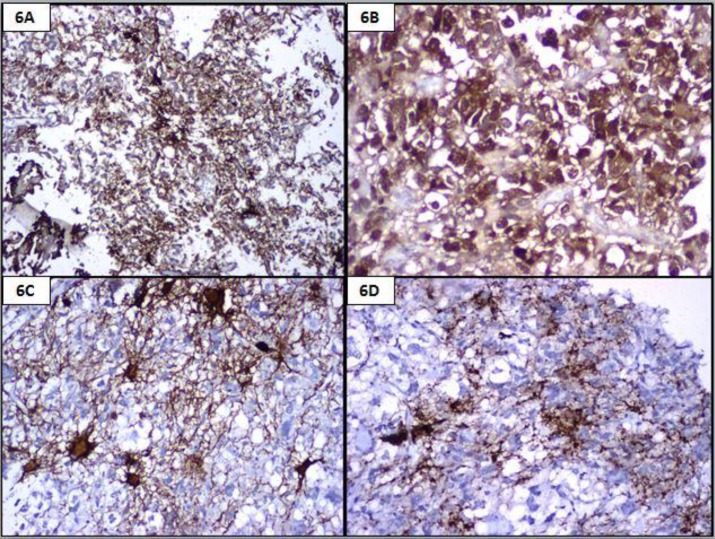
Positivity with GFAP (6A, IHC, 40x), S100 (6B, IHC, 400x), NSE (6C, IHC, 100x) and synaptophysin (6D, IHC, 100x)

**Figure 7:**
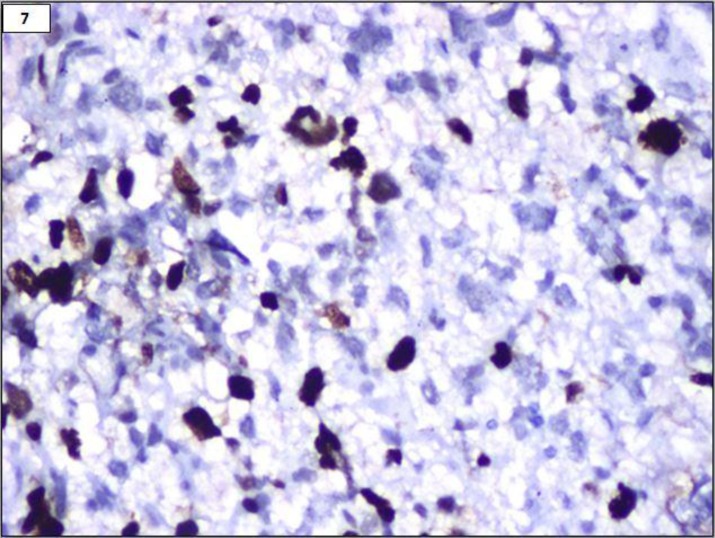
Ki67 labelling 18% (IHC, 400x)

An 18- year-old female presented with repeated vomiting, headache, weakness of right upper limb and slurring of speech. MRI showed a left fronto-parietal mass with midline shift. Fronto-parietal craniotomy and excision of the tumour were done with a pre-operative diagnosis of high grade glioma. Histomorphologic examination and immunohistochemistry findings are summarised in Table 2. A diagnosis of anaplastic pleomorphic xanthoastrocytoma was finally made (Figures 5, 6 and 7).

Two years post-operatively, she came back with similar complaints and a repeat mass in the same site was found. Repeat biopsy showed highly cellular tumour with moderate to marked nuclear atypia and pleomorphism, markedly increased mitoses of 1-2 per high power field (HPF), focal microvascular proliferation and necrosis. GFAP was found to be positive along with 40% Ki67 and strong p53 positive in more than 90% of tumour cells. A diagnosis of malignant transformation to glioblastoma in a known case of pleomorphic xanthoastrocytoma with features of anaplasia was made. After one-year follow-up, the patient showed spinal metastasis.

**Clinical follow- up**

All the patients were on routine follow-up after the standard modalities of treatment for more than one year post-operatively. They were all disease-free except one patient (Case 5) who developed a malignant transformation to glioblastoma (WHO grade IV) with evidence of spinal metastasis after one year post surgery. 

## Discussion

 PXA, since its first description in 1979, has received scant documentation in the medical literature. This holds true particularly for those with the features of anaplasia ^1^. It is known to be a tumour of the children, though exceptions are known ^2^^,^^4^^,^^6^.

Initially this entity was believed to be of astrocytic origin. However, of late, there has been a change in the understanding of the nature of these tumours. According to the available data, it has been proposed to be a developmental tumour of multipotent ectodermal origin, displaying neuronal differentiation and CD 34 positivity with a related, albeit focal, cortical dysplasia. The latter has been portrayed by the persistence of seizures despite complete surgery^7^^,^^8^. Based on immunohistochemical and electron microscopic studies by various authors, they seem to represent the connecting link between developmental abnormalities and ganglionic tumours of the well- differentiated type ^9^^,^^10^. There is a need to accurately diagnose this astrocytoma, for on one hand it can mimic other entities based on the location and morphology, posing a diagnostic dilemma, and, on the other hand, it can show anaplasia and aggressive behaviour in the form of recurrences and malignant transformation ^2^^,^^4^^,^^11^ . APXAs are believed to show the interphase between the lipidized giant-cell glioblastoma and PXA ^12^. 

Documented mortality in PXA ranges from 15 to 20%^13^. The anaplastic features have been found to be particularly of poor prognosis^5^. The latter has been described as those which show necrosis, cellular anaplasia which is severe, proliferating microvasculature, brisk mitosis (≥5 mitosis/10 high power fields) and increased Ki-67 labelling index^3^^,^^5^. As shown by the present study and those by Tonn et al., Sharma et al., and Ng et al., these patients typically present at a young age, though exceptional cases of elderly patients do occur ^4^^,^^6^^,^^14^. Contrary to the typical clinical presentation of PXAs with seizures and cortical lesions, the cases in this series of anaplastic PXAs highlight a gamut of varied presentation, ranging from space occupying lesions presenting with headache and weakness to paraplegia and incontinence with an intramedullary mass; the latter being exceedingly rare^15^. Radiologically, most of our cases showed vascular, dural-based and cortical masses with some cystic changes which were known to occur^11^.

Histologically, a biphasic tumour with moderate to high cellularity, pleomorphic and spindle cells, hyperchromatic nuclei showing moderate to marked atypia, intranuclear inclusions, 5-8 mitoses per 10 high-power field and foamy cytoplasm with focal evidence of necrosis and atypical mitoses were the usual pictures in most of our cases. The case showing a transformation into glioblastoma demonstrated a higher mitotic count of 8/10HPF in the primary tumour itself commensurate to the established criteria that *mitotic count* is the single most important predictor of the progression ^3^^, ^^8^^,^^16^. Eosinophilic granular bodies and lymphocytic infiltrates within the tumour were consistent findings. Similar morphologic observations were documented in other studies ^17^. Unlike other studies, there was no distinct plane identifiable between the tumour and the brain because of the brain invasion, which was a documented feature in most of our cases ^18^.

Immunohistochemistry of the tumours in this series showed a more or less consistent positivity for GFAP, S100, NSE and CD34 with a significant Ki67 expression. This tumour characteristically shows the expression of glial markers such as GFAP, S100 in most cases with simultaneous expression of neuronal markers Synaptophysin and class III beta-tubulin as well^19^. This dual differentiation has been documented in all the cases studied along with Ki-67 labelling index, ranging from 5-20%. The PXAs show Ki67 labelling in the range less than 1%, whereas the APXAs show expression from 5-10%, which helps distinguish PXA from APXA^19^. Morphologically, APXA is a close mimic of giant-cell glioblastoma. The expression of neuronal antigens, at least focally, by the individual tumour cells in PXA contrasts against the rare expression of neuronal polypeptides by glioblastoma, the latter shows strong p53 expression ^20^.

APXAs are rare tumours, so little is known about their molecular profile. BRAF V600E mutation is frequent in PXA and in 65% of APXA cases ^18^^,^^21^. The expression of CD34 has been correlated with BRAF mutation in cases of PXA which have at least a focal expression in most cases within the endothelial cells as well as dysplastic cortical cells ^21^. In the present series, two cases were associated with focal CD34 expression.

Primary APAXs have a shorter recurrence interval than secondary APXAs; however, the latter have poorer prognosis ^22^. All the five cases in the present study were primary APXA. Although one of the case studies showed a transformation into glioblastoma with evidence of spinal metastasis, all the patients are alive and on a routine postoperative follow-up. A complete surgical excision is required for a prolonged disease-free interval ^5^^,^^13^. Due to its rarity, the role of post-operative radiotherapy and chemotherapy is still not well established^1^^,^^5^.

Most of the information available about APXA comes from isolated case reports. This is one of the largest series of cases of anaplastic pleomorphic xanthoastrocytoma, compiled with an effort to understand the clinicoradiological, histopathological and immunohistochemical features. 

## CONCLUSION

 This study highlights the importance of diversity in the clinical presentation of APXA cases as a differential diagnosis in appropriate clinicoradiological settings to perform the relevant immunologic and/or molecular studies to exclude more aggressive mimics.
